# P-62. RSV Immunization Uptake and Barriers to Access Among Eligible Individuals During the First Season of Availability

**DOI:** 10.1093/ofid/ofae631.269

**Published:** 2025-01-29

**Authors:** Sarah E Smith-Jeffcoat, Sara Benist, Erin South, E Ivy Oyegun, J Bradford Bertumen, Carlos G Grijalva, Yuwei Zhu, Theresa A Scott, Jonathan Schmitz, Helen Y Chu, Ana A Weil, Janet A Englund, Melissa P MacMillan, Melissa Stockwell, Son H McLaren, Ellen Sano, Celibell Vargas, Keipp Talbot, Hannah L Kirking

**Affiliations:** Centers for Disease Control and Prevention, Atlanta, Georgia; Centers For Disease Control and Prevention, Atlanta, Georgia; Centers for Disease Control and Prevention, Atlanta, Georgia; Centers for Disease Control and Prevention, Atlanta, Georgia; Centers for Disease Control and Prevention, Atlanta, Georgia; Vanderbilt University Medical Center, Nashville, Tennessee; Vanderbilt University, Nashville, Tennessee; Vanderbilt University Medical Center, Nashville, Tennessee; Vanderbilt University Medical Center, Nashville, Tennessee; University of Washington, Seattle, WA; University of Washington, Seattle, WA; Seattle Children’s Hospital, Seattle, Washington; University of Washington, Seattle, WA; Columbia University Irving Medical Center, New York City, New York; Columbia University Irving Medical Center, New York City, New York; Columbia University Irving Medical Center, New York City, New York; Columbia University Irving Medical Center, New York City, New York; Vanderbilt University Medical Center, Nashville, Tennessee; Coronavirus and Other Respiratory Viruses Division, National Center for Immunization and Respiratory Diseases, CDC, Atlanta, GA

## Abstract

**Background:**

By fall 2023, RSV immunizations to protect older adults, pregnant people, and infants were recommended in the United States. Our aim was to describe uptake of and barriers to RSV immunization among community-based individuals during the first season of availability.

**Table 1:**
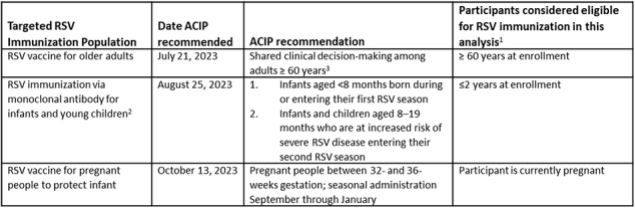
Targeted RSV immunization populations, ACIP recommendations, and participant eligibility criteria for this analysis, April 2024 1. Due to limitations in how data were collected in the parent study, the eligibility criteria used for the analysis may differ from exact ACIP recommended criteria. 2. Nirsevimab, a single injection during the infant’s first respiratory season, was recently recommended by ACIP and preferred by American Academy of Pediatrics over palivizumab, which requires monthly injections during the respiratory season and is recommended for a subset of high-risk infants. Palivizumab has been available in the United States since 1998. 3. Shared clinical decision-making allows flexibility for providers and patients to consider individual risk for RSV disease, while considering patient preferences. RSV = respiratory syncytial virus; ACIP = Advisory Committee for Immunization Practices

**Methods:**

Starting January 2024, participants were enrolled in a household transmission study of COVID-19 and RSV. All participants completed questionnaires capturing demographics, household characteristics, vaccination and medical history, and their knowledge, attitudes, and behaviors towards RSV and RSV immunizations. Participants included in this analysis were those eligible for RSV immunizations based on Advisory Committee for Immunization Practices guidelines (Table 1). Participants were considered immunized if they had a verified RSV immunization or plausible self-report i.e., reported an RSV immunization with the date of the immunization and a location or manufacturer (Figure 1). We compared characteristics, knowledge, attitudes, and behaviors stratified by immunization uptake using chi-squared or Fishers exact tests.

**Figure 1:**
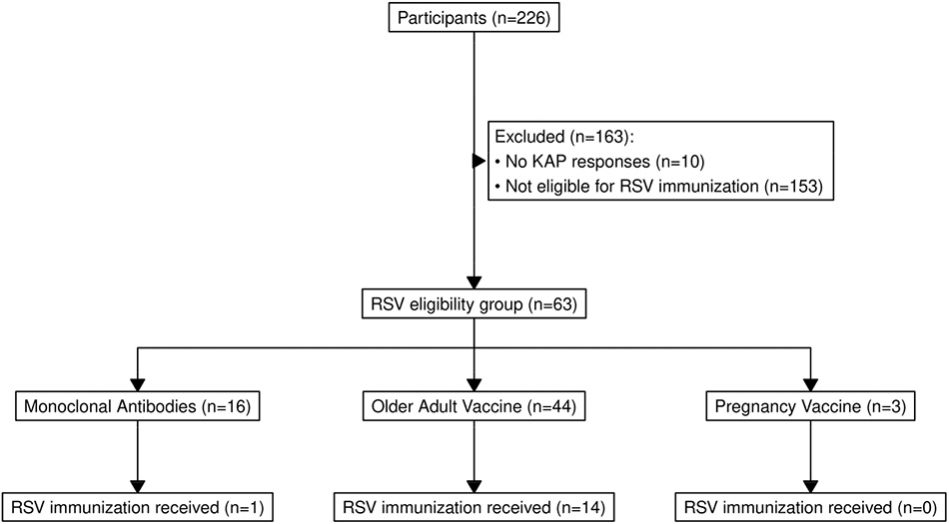
Participant RSV immunization eligibility and receipt1 1. Immunization history was self-reported by participants and verified by study staff using medical and pharmacy records. Participants were considered immunized if they had a verified RSV immunization or plausible self-report meaning they self-reported an RSV immunization with the date of the immunization and a location or manufacturer. RSV = respiratory syncytial virus; KAP = Knowledge, Attitudes, and Practices survey

**Results:**

Among participants enrolled and eligible for this analysis as of April 2024 (n=63), 15 (24%) received RSV immunization: 14/44 (32%) among older adults, 1/16 (6%) among infants/young children, and 0/3 (0%) among pregnant people (Figure 1). Older adults who did not receive the vaccine were more likely to be younger (67 vs 71 years, p=0.009) and less likely to have received an updated 2023-24 COVID-19 vaccine (40% vs 79%, p=0.02; Table 2). Among older adults who replied they would not or were not sure if they would get vaccinated, reasons included that they needed more information (43%; 9/21) or were not sure how/where to get the vaccine (14%, 3/21; Table 3).

**Table 2:**
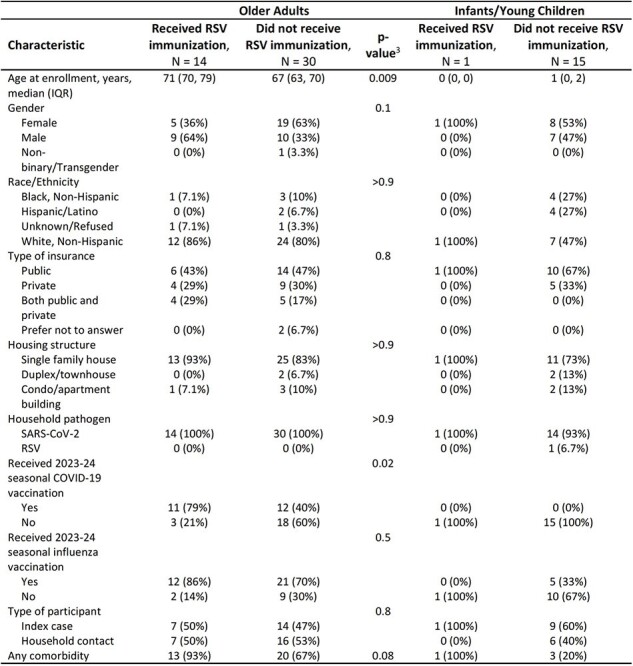
Individual and household characteristics of participants eligible for RSV immunization1 by RSV immunization status2 1 RSV immunization for older adults is a vaccine and for infants/young children is a monoclonal antibody 2 Immunization history was collected self-reported by participants and verified by study staff using medical and pharmacy records. Participants were considered immunized if they had a verified RSV immunization or plausible self-report meaning they self-reported an RSV immunization with the date of the immunization and a location or manufacturer. 3 Wilcoxon rank sum test; Fisher's exact test; Pearson's Chi-squared test mAb = monoclonal Antibodies; RSV = respiratory syncytial virus

**Conclusion:**

A quarter of individuals who were eligible for RSV immunization had received it during the 2023-24 RSV season. Older adults on the younger end of the recommendation were less likely to be vaccinated; it is unclear if this is due to limited awareness of RSV disease, perceived lower risk, or other reasons. Better education and awareness of RSV disease and vaccine safety, effectiveness, and availability may increase uptake among older adults. Ongoing enrollment may allow insight into barriers for infants and pregnant people.

Table 3: Individual beliefs and attitudes towards RSV and RSV immunizations of participants eligible for RSV immunization1 by RSV immunization status2

**Figure:**
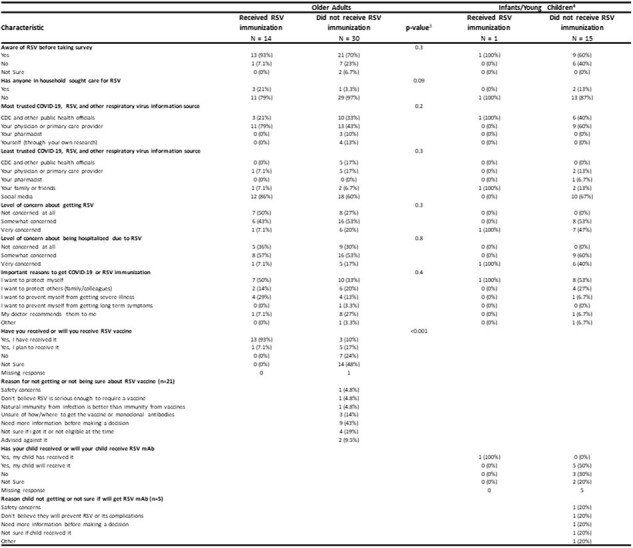

1 RSV immunization for older adults is a vaccine and for infants/young children is a monoclonal antibody

2 Immunization history was collected self-reported by participants and verified by study staff using medical and pharmacy records. Participants were considered immunized if they had a verified RSV immunization or plausible self-report meaning they self-reported an RSV immunization with the date of the immunization and a location or manufacturer.

3 Fisher's exact test

4 Responses provided by a parent/caregiver of infant/young child

**Disclosures:**

**Carlos G. Grijalva, MD, MPH**, AHRQ: Grant/Research Support|CDC: Grant/Research Support|FDA: Grant/Research Support|Merck: Advisor/Consultant|NIH: Grant/Research Support|SyneosHealth: Grant/Research Support **Jonathan Schmitz, MD, PhD, D(ABMM)**, Biofire/Biomerieux: Grant/Research Support|Genmark/Roche: Grant/Research Support|Pfizer: Honoraria|Vela: Grant/Research Support **Helen Y. Chu, MD, MPH**, Abbvie: Advisor/Consultant|Merck: Advisor/Consultant|Vir: Advisor/Consultant **Janet A. Englund, MD**, Abbvie: Advisor/Consultant|AstraZeneca: Advisor/Consultant|AstraZeneca: Grant/Research Support|GlaxoSmithKline: Advisor/Consultant|GlaxoSmithKline: Grant/Research Support|Meissa Vaccines: Advisor/Consultant|Merck: Advisor/Consultant|Pfizer: Board Member|Pfizer: Grant/Research Support|Pfizer: Speaker at meeting|SanofiPasteur: Advisor/Consultant|Shinogi: Advisor/Consultant

